# Supplementary material for: Revealing a New Family of D-2-Hydroxyglutarate Dehydrogenases in Escherichia coli and Pantoea ananatis Encoded by ydiJ
Source: Microorganisms. 2022 Aug 31;10(9):1766. doi: 10.3390/microorganisms10091766 (PMC9504171; doi:10.3390/microorganisms10091766)
Supplement: Supplementary file 1 [file microorganisms-10-01766-s001.zip › Supplementary.pdf]

Table S1. Primers used in this study

Primer	Sequence	Description
ydiJ_EcF1	ATGATTCGAAC**ATG**ATTCCACAGATTTCCCAG<br>*Bsp119I*	Forward primer for *ydiJ* from *E. coli*
ydiJ_EcR1	ATATCTCGAGTCATTTAATAATCTCCAGTAA<br>*XhoI*	Reverse primer for *ydiJ* from *E. coli*
ydiJ_EcF2	ATATCC**ATG**GTTCCACAGATTTCCCAGGCACC<br>*NcoI*	Forward primer for *ydiJ* from *E. coli*
ydiJ_EcR2	ATATGGATCCCCTTTAATAATCTCCAGTAAAGCCTG<br>*BamHI*	Reverse primer for *ydiJ* from *E. coli*
ydiJ_PaF1	TAGTGACAT**ATG**ATCCCACAGATTTCTCAGGCACC<br>*NdeI*	Forward primer for *ydiJ* from *P. ananatis*
ydiJ_PaR1	GTGACTCGAGGCGTCGCCTCACCTTTTCAGATT<br>*XhoI*	Reverse primer for *ydiJ* from *P.ananatis*
ydiJ_PaF2	ATTAAC**ATG**TTCCCACAGATTTCTCAGGCACC<br>*PscI*	Forward primer for *ydiJ* from *P. ananatis*
ydiJ_PaR2	GTGACTCGAGGATTAACTCAAGCAGTGCCTGAAG<br>*XhoI*	Reverse primer for *ydiJ* from *P. ananatis*
ydiJ_PaR_D	GGATGACGCATGCCGTTGCCCTCCACGCGCTTAACC TGAAGCCTGCTTTTTTATACTAAGTTGG	Reversed primer for deletion of *ydiJ* from *P. ananatis*
ydiJ_PaF_D	TCCCACAGATTTCTCAGGCACCGGGTCTTATCCAGC CGCTCAAGTTAGTATAAAAAAGCTGAAC	Forward primer for deletion of *ydiJ* from *P. ananatis*
ydiJ_PaF_P8	GCGTTTAAACAGGCTTGTAAAGAAAAGGTTTCAGC CTGAAGCCTGCTTTTTTATACTAAGTTGG	Forward primer for replacement of wild-type P_{ydiJ} promoter by P_{nlp8} upstream of gene $ydiJ_{Pa}$
ydiJ_PaR_P8	GATAAGACCCGGTGCCTGAGAAATCTGTGGGATCA TTTATCCCCCAGGAAAAATTGGTTA	Reverse primer for replacement of wild-type P_{ydiJ} promoter by P_{nlp8} upstream of gene $ydiJ_{Pa}$
serA_EcF	AAGCTTGCATGCATTCCAACTGCGCTAATGACGC<br>*SphI*	Forward primer for construction of pMIV-P_{nlp8}-serA348(Ec) expression plasmid
serA_EcR	CTGTTTTCTAGAGCGTGGGATCAGTAAAGCAGGC<br>*XbaI*	Reverse primer for construction of pMIV-P_{nlp8}-serA348(Ec) expression plasmid
serA_EcF_aux	AACCAATTTTTCCTGGGGGTCGACATGGCAAAGGT ATCACTGGAAAAAGAC *SalI*	Forward auxiliary primer for pMIV-P_{nlp8}-serA348(Ec) construction
serA_EcR_aux	GTCTTTTTCCAGTGATACCTTTGCC**ATG**TCGACCCCC AGGAAAAATTGGTT *SalI*	Reverse auxiliary primer for pMIV-P_{nlp8}-serA348(Ec) construction
ydiJ_EcF_P8	GCTGCGGCTACGTTCGTTCACCCCAGTCACTTACTA TGAAGCCTGCTTTTTTATACTAAGTTGG	Forward primer for replacement of wild-type P_{ydiJ} promoter by P_{nlp8} upstream of gene $ydiJ_{Ec}$
ydiJ_EcR_P8	AACGACGCCGGGTGCCTGGGAAATCTGTGGAATCA TTTATCCCCCAGGAAAAATTGGTTA	Reverse primer for replacement of wild-type P_{ydiJ} promoter by P_{nlp8} upstream of gene $ydiJ_{Ec}$
ydiJ_EcF_D	TTCCACAGATTTCCCAGGCACCCGGCGTCGTTCAAC CGCTCAAGTTAGTATAAAAAAGCTGAAC	Forward primer for deletion of *ydiJ* from *E. coli*
ydiJ_EcR_D	ACAGGATGGCGTACCCCCGTGCCTTCAACCCGTTTT TGAAGCCTGCTTTTTTATACTAAGTTGG	Reverse primer for deletion of *ydiJ* from *E. coli*

Recognition sites for restriction endonucleases are underlined, start codons are in bold

Table S2. Distribution of YdiJ-like enzymes with identity to YdiJEc more than 50% among Proteobacteria filum (taxid_1224) constructed by means of the NCBI BLASTP service (Database: Reference proteins (refseq_protein))

Table S3. Taxonomy presentation of distribution of YdiJ-like enzymes with identity to YdiJEc more than 50% among Proteobacteria filum (taxid_1224) constructed by means of the NCBI BLASTP service (Database: Reference proteins (refseq_protein))

Figure S1. Lineweaver–Burk plot for the purified D2HGDHPa (A) and D2HGDHEc (B) toward D-2-HGA. Activity of D2HGDHs was measured as described in "Materials and methods" at variable concentrations of D-2-HGA at optimum pH. Data shown are mean ± s.d. (n = 3 independent experiments)

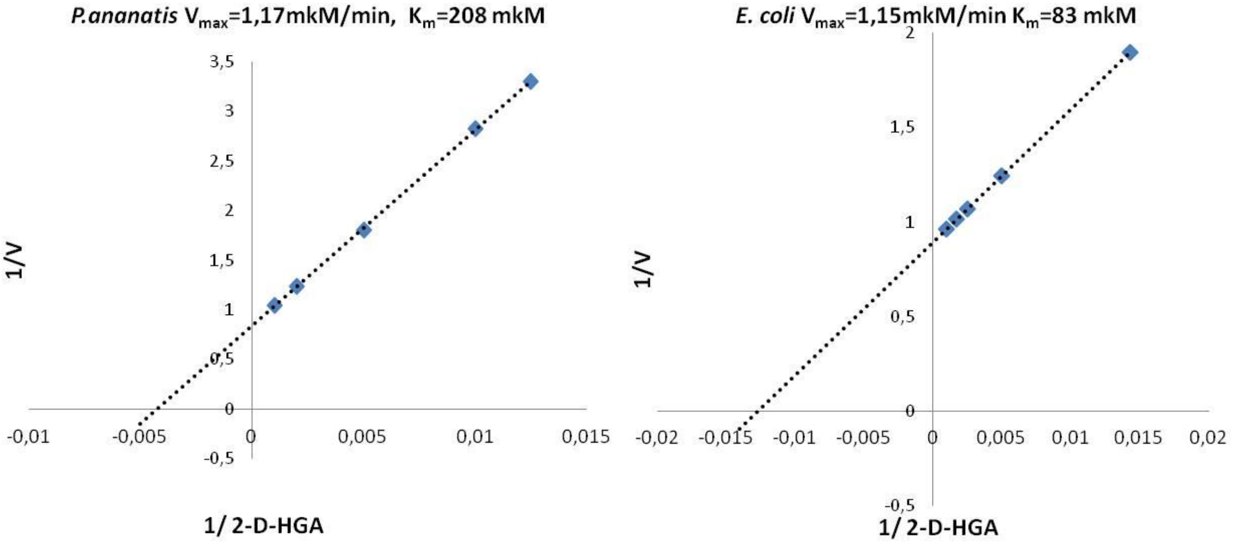
P.ananatis V_{max}=1,17mkM/min, K_m=208 mkM
1/V
1/ 2-D-HGA
E. coli V_{max}=1,15mkM/min K_m=83 mkM
1/V
1/ 2-D-HGA

A B
