# Supplementary figures and images for: Revealing a New Family of D-2-Hydroxyglutarate Dehydrogenases in Escherichia coli and Pantoea ananatis Encoded by ydiJ
Source: Microorganisms. 2022 Aug 31;10(9):1766. doi: 10.3390/microorganisms10091766 (PMC9504171; doi:10.3390/microorganisms10091766)

*P. ananatis*  $V_{\max}=1,17\text{mkM}/\text{min}$ ,  $K_m=208\text{ mkM}$

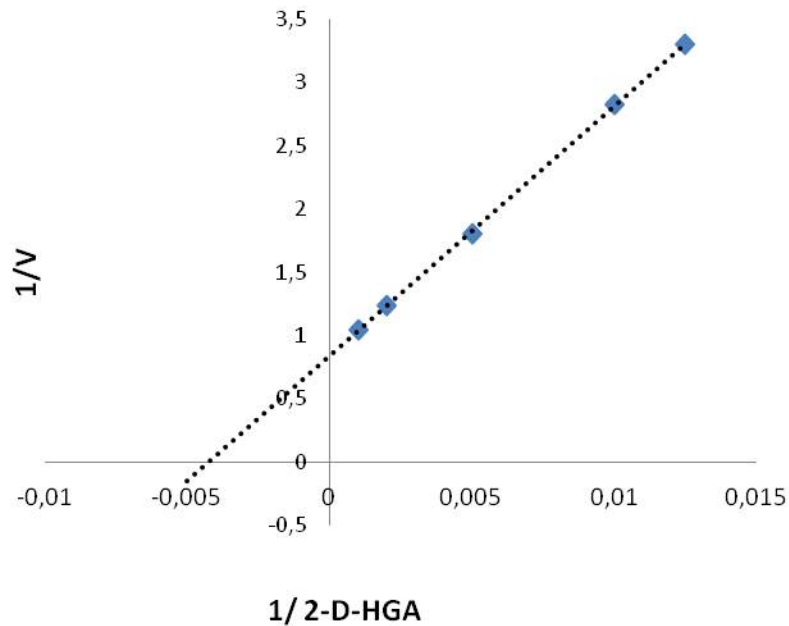

A

*E. coli*  $V_{\max}=1,15\text{mkM}/\text{min}$ ,  $K_m=83\text{ mkM}$

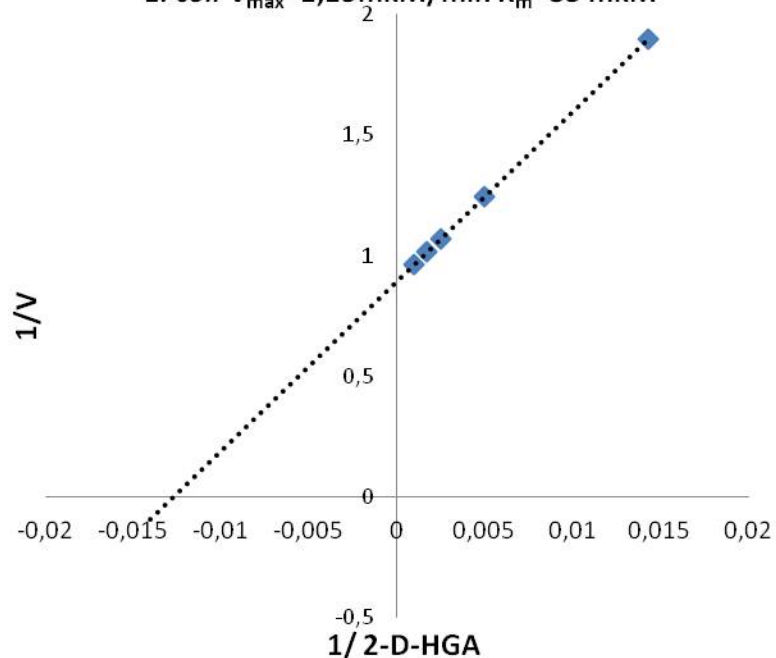

B

Supplement: Supplementary file 1 [file microorganisms-10-01766-s001.zip › Figure S1.pdf]
